# Does Pemetrexed Work in Targetable, Nonsquamous Non-Small-Cell Lung Cancer? A Narrative Review

**DOI:** 10.3390/cancers12092658

**Published:** 2020-09-17

**Authors:** Jin-Yuan Shih, Akira Inoue, Rebecca Cheng, Rocio Varea, Sang-We Kim

**Affiliations:** 1 Department of Internal Medicine, National Taiwan University Hospital, No. 7 Zhongshan South Road, Zhongzheng District, Taipei City 100, Taiwan; 2 Graduate Institute of Clinical Medicine, National Taiwan University, 7 Chung-Shan S. Rd., Taipei 100, Taiwan; 3 Department of Palliative Medicine, Tohoku University School of Medicine, 2-1 Seiryo-machi, Aoba-ku, Sendai, Miyagi 980-8575, Japan; akira.inoue.b2@tohoku.ac.jp; 4 Eli Lilly and Company, Songshan District, Fuxing North Road 365, Taipei 105, Taiwan; cheng_rebecca@lilly.com (R.C.); varea_menendez_rocio@lilly.com (R.V.); 5 Department of Oncology, Asan Medical Center, 88 Olympic-Ro 43-Gil, Songpa-Gu, Seoul 05505, Korea; swkim@amc.seoul.kr

**Keywords:** non-small-cell lung cancer, gain of function mutation, chemotherapy, pemetrexed, progression-free survival

## Abstract

**Simple Summary:**

The chemotherapy agent pemetrexed is currently considered in combination with other therapies for the treatment of advanced nonsquamous non-small-cell lung cancer (NSCLC) in patients negative for gene mutations/rearrangements. The aim of this review was to highlight data from clinical studies with pemetrexed in patients with advanced nonsquamous NSCLC positive for gene mutations/rearrangements. The results of the review suggest that pemetrexed could be a treatment option in patients with advanced nonsquamous NSCLC positive for certain gene mutations/rearrangements.

**Abstract:**

Pemetrexed is currently mainly considered for the treatment of advanced nonsquamous non-small-cell lung cancer (NSCLC) negative for gene mutations/rearrangements (wild-type disease (WTD)). This narrative review aimed to highlight the role of pemetrexed in the treatment of onco-driven nonsquamous advanced NSCLC by reviewing published clinical studies. For epidermal growth factor receptor (EGFR) mutations, patient survival following first-line pemetrexed–platinum was longer than for WTD. Later-line pemetrexed-based treatment after tyrosine kinase inhibitor (TKI) failure provided greater benefits than non-pemetrexed regimens. First- and later-line pemetrexed-based therapy also provided survival benefits in patients with anaplastic lymphoma kinase (ALK) or ROS proto-oncogene 1 (ROS1) rearrangements. In patients with rearranged during transfection (RET) proto-oncogene rearrangements, survival with pemetrexed was similar to that in ALK- and ROS1-positive patients and longer than that in patients with Kirsten rat sarcoma (KRAS) virus proto-oncogene mutations or WTD, although the available studies were limited. For Erb-b2 receptor tyrosine kinase 2 (ERRB2) mutations, first-line pemetrexed showed outcomes similar to those for EGFR and KRAS alterations. Data on pemetrexed in patients with KRAS mutations or MNNG HOS-transforming (MET) expression were limited. Pemetrexed could be an option for first- and second-line treatment for TKI failure in nonsquamous advanced NSCLC with select targetable driver mutations.

## 1. Introduction

Non-small-cell lung cancer (NSCLC) accounts for more than 80% of lung cancer cases [1] and is the leading cause of death from cancer worldwide [2]. Most patients with NSCLC are diagnosed with advanced-stage disease. Within the past decade, several gene mutations and rearrangements in NSCLC have been identified, leading to the development of treatments specifically targeting these molecular alterations (Table 1) [3,4,5,6]. It is currently recommended that all patients with nonsquamous NSCLC be tested for certain genetic alterations and the results used to guide treatment [7].

Pemetrexed is an antifolate cytotoxic agent with a broad spectrum of antitumor activity. It works by inhibiting multiple folate-dependent enzymes (e.g., thymidylate synthase (TS), glycinamide ribonucleotide formyl transferase, and dihydrofolate reductase), which are essential for cell replication [8]. Pivotal trials of pemetrexed in advanced NSCLC patients have confirmed that, in combination with platinum, pemetrexed provides survival benefit as a first-line treatment [9], as does pemetrexed monotherapy as maintenance or second-line treatment [10,11,12]. However, the benefits of pemetrexed are confined to patients with nonsquamous histology [9,11] (Supplementary Table S1). TS expression is significantly higher in squamous cell carcinoma than in adenocarcinoma [13], and multiple cell-line studies suggest that high TS expression is associated with reduced sensitivity to pemetrexed [14,15]; although this has not been validated clinically, it may partly explain the superior efficacy of pemetrexed in nonsquamous versus squamous NSCLC [16].

Epidermal growth factor receptor (EGFR) mutations and anaplastic lymphoma kinase (ALK) rearrangements are common in NSCLC and are associated with low levels of tumor cell TS expression [17], suggesting that pemetrexed would be consistently effective against nonsquamous NSCLC with these genomic alterations. Indeed, multiple studies have shown that pemetrexed is effective against nonsquamous NSCLC with targetable driver mutations [18,19,20,21,22,23,24,25,26,27,28,29,30,31,32,33,34,35,36,37,38,39,40,41,42,43,44,45,46,47,48,49,50,51,52,53,54,55].

Guidelines generally recommend pemetrexed in combination with carboplatin or cisplatin and immunotherapy for first-line treatment and as monotherapy for later-line treatment of advanced nonsquamous NSCLC in patients with a performance status of 0/1 who are negative for gene mutations/rearrangements (i.e., with wild-type disease; Supplementary Table S2) [3,4,56,57]. Since 2018, the European Society of Medical Oncology (ESMO) has recommended pemetrexed plus carboplatin and gefitinib as first-line treatments in patients with an EGFR-activating mutation [4,56].

The aim of this narrative review is to highlight the role of pemetrexed in the treatment of targetable nonsquamous advanced NSCLC by reviewing clinical outcomes with the drug in published clinical studies, including systematic reviews, randomized controlled trials, prospective cohort studies, post-hoc analyses, and retrospective chart reviews. Relevant studies were identified from nonsystematic searches of PubMed using the search terms “NSCLC” and “pemetrexed” and “EGFR”, “ALK”, “ROS1”, “RET”, “ERBB2”, “HER2”, “KRAS”, or “MET”. Searches were performed on 18 February 2019.

## 2. EGFR Mutations

### 2.1. First-Line Treatment

#### 2.1.1. Pemetrexed–Platinum

Patients with EGFR mutations treated with first-line pemetrexed–platinum have shown better progression-free survival (PFS) and overall response rate (ORR) than patients with wild-type disease. In a retrospective cohort study of pemetrexed-based chemotherapy in patients with EGFR mutations (n = 69) or wild-type disease (n = 89), median PFS (8.3 versus 6.7 months, respectively; *p* = 0.004) and ORR (43 versus 21%, respectively; *p* = 0.039) were significantly greater in EGFR-positive than in wild-type patients for those receiving pemetrexed–platinum (Supplementary Table S3) [18].

**Figure 1:**
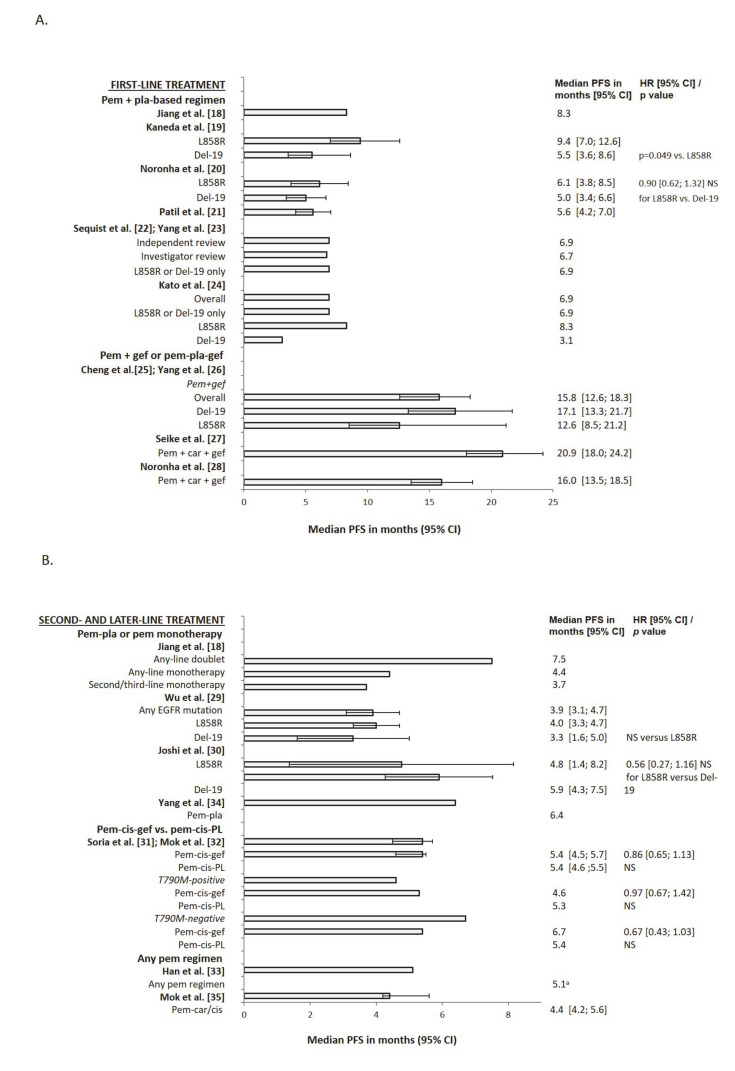
Median progression-free survival (PFS) with (**A**) first-line and (**B**) second- and later-line pemetrexed-based treatment in patients with advanced nonsquamous NSCLC and EGFR gene mutations. ^a^ Weighted median PFS. Abbreviations: carboplatin (car); cisplatin (cis); confidence interval (CI); EGFR gene exon 19 deletion (Del-19); epidermal growth factor receptor (EGFR); gefitinib (gef); hazard ratio (HR); Leu858Arg point mutation in exon 21 (L858R); not significant (NS); non-small-cell lung cancer (NSCLC); pemetrexed (pem); progression-free survival (PFS); placebo (PL); platinum (pla).

The influence of EGFR mutation type on the benefits of pemetrexed therapy is unclear. In a retrospective chart review of 304 patients receiving first-line pemetrexed–cisplatin, median PFS was significantly longer in patients with Leu858Arg point mutations in exon 21 (L858R mutations (n = 42): 9.4 months) than in those with exon 19 deletions (Del-19 mutations (n = 36): 5.5 months; *p* = 0.049) (Figure 1). However, neither median overall survival (OS) nor ORR differed significantly between the two groups (Supplementary Table S3). In a multivariate analysis, only EGFR mutation status significantly predicted prolonged PFS (hazard ratio (HR) 0.78; *p* = 0.033) [19].

By contrast, a post-hoc analysis of a phase III open-label study of pemetrexed–carboplatin found no significant difference in median PFS or ORR between patients with L858R (n = 51) or Del-19 mutations (n = 92) (Figure 1 and Supplementary Table S3). However, median OS was significantly longer in Del-19-positive (24.5 months) than in L858R-positive patients (18.1 months; *p* = 0.002). In a multivariate analysis, EGFR mutation type significantly predicted longer OS (HR 0.43; *p* = 0.001) [20].

#### 2.1.2. Pemetrexed–Platinum Versus TKIs

In studies comparing first-line pemetrexed–platinum or tyrosine kinase inhibitor (TKI) therapy in EGFR-positive patients, median PFS and ORR were greater with TKI therapy. In an open-label study comparing gefitinib (n = 145) with pemetrexed–carboplatin induction followed by maintenance pemetrexed (n = 145), median PFS and ORR were significantly greater with gefitinib than with chemotherapy (median PFS 8.4 months versus 5.6 months, respectively; HR 0.66; *p* = 0.001; ORR 63.5 versus 45.3%, respectively; *p* = 0.003) (Supplementary Table S3). OS was similar for the two treatments (18.0 versus 22.6 months, respectively; HR 1.28; not significant (NS)) [21].

Similarly, a phase III study (LUX-Lung 3) of 345 EGFR-positive patients (L858R, Del-19 or other) who received afatinib (n = 230) or pemetrexed–cisplatin (n = 115) showed that afatinib significantly prolonged median PFS versus chemotherapy (11.1 versus 6.9 months, respectively; HR 0.58; *p* = 0.001) [22] (Supplementary Table S3). The difference between afatinib and pemetrexed–cisplatin was even greater for common EGFR mutations (L858R or Del-19 mutations only (n = 308): median PFS 13.6 versus 6.9 months, respectively; HR 0.47; *p* = 0.001). ORR was also significantly greater for afatinib versus chemotherapy (56 versus 23%; *p* = 0.001), but OS was similar (28.2 months for both; *p* = 0.39) [23]. Comparable results were obtained in a subgroup analysis of LUX-Lung 3 Japanese patients [24].

#### 2.1.3. Pemetrexed–Gefitinib Versus Gefitinib

Adding gefitinib to pemetrexed or pemetrexed–platinum improved PFS to a greater extent than treatment with gefitinib alone in first-line settings in EGFR-positive patients. In a phase II open-label study comparing pemetrexed–gefitinib (n = 126) with gefitinib monotherapy (n = 65), median PFS was significantly prolonged in patients receiving combination therapy (15.8 months) versus those receiving gefitinib alone (10.9 months; adjusted HR 0.68; two-sided *p* = 0.029) (Supplementary Table S3). An analysis according to EGFR mutation type (L858R or Del-19; patient numbers not given) showed a significant improvement in PFS with combination therapy versus gefitinib for both mutation types [25]. ORR (80% versus 74%, respectively) and OS (median 43.4 versus 36.8 months, respectively; HR 0.77; one-sided *p* = 0.105) did not differ between the two treatments [26]. Similar results were obtained in two phase III open-label studies for median PFS, but OS was significantly longer with combination therapy (Supplementary Table S3) [27,28].

### 2.2. Second- and Later-Line Treatment

#### 2.2.1. Pemetrexed–Platinum or Pemetrexed Monotherapy

In the second- or later-line setting, median PFS and ORR were numerically or significantly greater in EGFR-positive than in wild-type patients for pemetrexed–platinum doublet therapy and pemetrexed monotherapy. A previously mentioned retrospective study [18] evaluated all lines of pemetrexed-based chemotherapy in patients with and without EGFR mutations (pemetrexed–platinum: n = 96; pemetrexed monotherapy: n = 62). For any-line doublet treatment, median PFS was 7.5 versus 6.4 months for EGFR-positive and wild-type patients, respectively (NS) (Supplementary Table S3). A similar pattern was observed for any-line monotherapy (4.4 versus 3.7 months; NS) and second-/third-line monotherapy (3.7 versus 3.2 months; NS). ORR was 27 versus 13% (NS) for second-line doublet therapy and 13 versus 8% (NS) for second-/third-line monotherapy.

In a prospective cohort study, median PFS was significantly longer in EGFR-positive (n = 93: 3.9 months) than in wild-type patients (n = 63: 2.3 months; *p* = 0.03) for second- or later-line pemetrexed therapy (Supplementary Table S3). However, there was no significant difference between mutation types (Del-19 (n = 43): 3.3 months; L858R (n = 37): 4.0 months; NS) (Figure 1). In a multivariate analysis, EGFR mutation (versus wild-type disease) was significantly associated with PFS (HR 0.68; *p* = 0.021). ORR was also significantly greater in EGFR-positive than in wild-type patients (12.9 versus 1.6%; *p* = 0.016) and did not vary with line of treatment (8.8%, 8.6%, and 7.8% for second-, third-, and fourth-line therapy, respectively; NS) or type of mutation (14.0% for Del-19 versus 10.8% for L858R mutation; NS; 12.5% for Del-19 or L858R mutation versus 15.4% for nonclassic mutation; NS). However, OS did not differ significantly between EGFR-positive and wild-type disease (30.8 versus 25.8 months) or between Del-19 and L858R mutations (34.1 versus 29.3 months) [29].

Another study also suggested that median PFS did not differ significantly between Del-19- (n = 33) and L858R-positive patients (n = 22) who progressed on first-line gefitinib and received second-line pemetrexed–carboplatin. Median PFS was 5.9 versus 4.8 months for Del-19- and L858R-positive patients, respectively (HR 0.56; NS) (Figure 1 and Supplementary Table S3). However, median OS was significantly longer in Del-19 (11.8 months) than in L858R patients (6.2 months; *p* = 0.024). In a multivariate analysis, EGFR mutation type did not significantly predict OS (HR 0.36; NS). ORR also did not differ significantly between the two mutation groups (L858R: 33.3%; Del-19: 39.3%) [30].

#### 2.2.2. Pemetrexed–Cisplatin–Gefitinib Versus Pemetrexed–Cisplatin–Placebo

Adding gefitinib to second-line pemetrexed–platinum did not further improve PFS in patients with EGFR-positive advanced NSCLC resistant to first-line gefitinib. In a phase III study (IMPRESS), median PFS was similar for patients receiving pemetrexed*–*cisplatin–gefitinib (n = 133) or pemetrexed*–*cisplatin–placebo (n = 132) (5.4 months for both: HR 0.86; NS), as was ORR (31.6 versus 34.1%, respectively; odds ratio (OR) 0.92; NS) [31]. In a further analysis of IMPRESS, OS was significantly longer with pemetrexed*–*cisplatin*–*placebo (19.5 versus 13.4 months with pemetrexed*–*cisplatin*–*gefitinib; HR 1.44; *p* = 0.016). An analysis according to T790M mutation status showed that median PFS did not differ significantly between the two treatments for either T790M-positive or T790M-negative patients, whereas OS was significantly longer in the pemetrexed*–*cisplatin*–*placebo group in T790M-positive patients (Figure 1 and Supplementary Table S3) [32].

#### 2.2.3. Pemetrexed Versus Non-Pemetrexed Regimens

Following TKI failure, the PFS benefit with second-line pemetrexed-based regimens is greater than that with non-pemetrexed-based regimens in EGFR-positive patients. In a systematic review of eight studies involving 640 patients receiving pemetrexed and 97 receiving non-pemetrexed regimens, weighted median PFS was longer for pemetrexed (5.1 months) than for non-pemetrexed regimens (3.2 months) (Supplementary Table S3). Median PFS was significantly longer for pemetrexed versus non-pemetrexed regimens in two of the three studies comparing these regimens (4.2 versus 2.7 months, HR 0.54; *p* = 0.009 in one study; 6.4 versus 4.1 months, HR 0.47; *p* = 0.01 in the other study), but not in the third (4.7 versus 3.3 months (NS)) [33].

Similar results were obtained for weighted median OS and weighted ORR. Weighted OS was longer for pemetrexed (15.9 months) than for non-pemetrexed regimens (11.1 months), with numerical but nonsignificant differences reported in the three studies comparing these regimens (15.1 versus 11.0 months, HR 0.92; 19.2 versus 14.1 months, 0.50; 15.1 versus 8.1 months; all *p*-values NS) (Supplementary Table S3). Weighted ORR was 30.2% for pemetrexed and 18.3% for non-pemetrexed regimens. Again, the differences reported in the two studies comparing these regimens were numerically but not significantly higher with pemetrexed versus non-pemetrexed regimens (32.4 versus 17.4% and 26.0 versus 20.0%, both *p*-values NS).

A retrospective cohort study evaluated second-line therapies in patients resistant to first-line gefitinib. Treatments included erlotinib monotherapy and platinum-based doublet therapy with pemetrexed, gemcitabine, vinorelbine, or taxanes. Median PFS was significantly longer for pemetrexed–platinum (n = 34) than for non-pemetrexed platinum-based doublet therapy (n = 26; 6.4 versus 4.1 months, *p* = 0.008; HR 0.42; 95% CI 0.23–0.77) (Supplementary Table S3). OS and ORR were numerically but not significantly greater with pemetrexed–platinum than with non-pemetrexed-based therapy (19.2 versus 14.1 months and 24 versus 12%, respectively; NS for both) [34].

#### 2.2.4. Pemetrexed–Platinum Versus Osimertinib

In a phase III open-label study (AURA3) median PFS and ORR were significantly greater with osimertinib than with pemetrexed*–*platinum in patients with EGFR-T790M-positive NSCLC. Among 419 EGFR-T790M-positive patients with disease progression after first-line TKI therapy, median PFS was 10.1 months with osimertinib versus 4.4 months with pemetrexed*–*platinum (HR 0.30; *p* < 0.001). ORR was 71 versus 31% (OR 5.39; *p* < 0.001) (Supplementary Table S3). OS data were immature and therefore not presented [35].

## 3. ALK Rearrangements

### 3.1. First-Line Treatment

#### 3.1.1. Pemetrexed–Platinum

**Figure 2:**
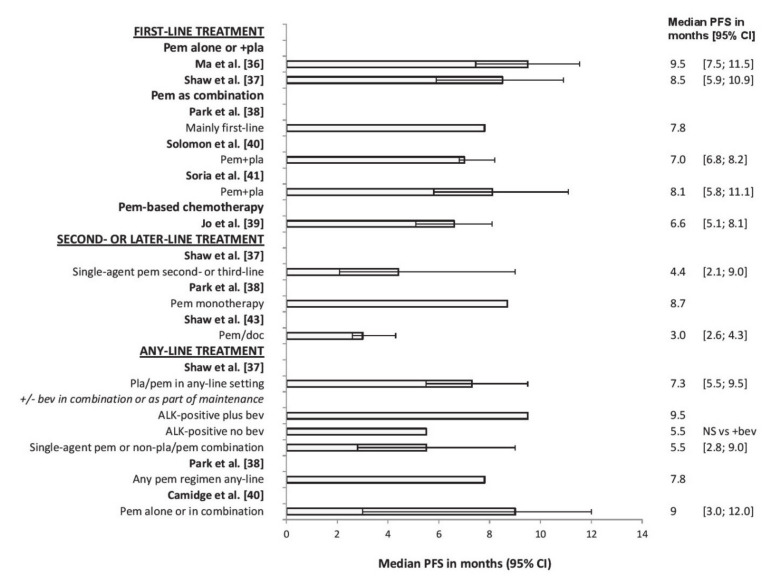
Median PFS with pemetrexed-based treatment in patients with advanced nonsquamous NSCLC and ALK rearrangements. Abbreviations: anaplastic lymphoma kinase (ALK); bevacizumab (bev); carboplatin (car); cisplatin (cis); confidence interval (CI); non-small-cell lung cancer (NSCLC); not significant (NS); pemetrexed (pem); progression-free survival (PFS); placebo (PL); platinum (pla); docetaxel (doc); hazard ratio (HR).

Studies have suggested that pemetrexed–platinum doublet therapy is effective in the first-line treatment of ALK-positive NSCLC. A retrospective chart review of 52 ALK-positive patients receiving first-line pemetrexed–platinum (two patients received single-agent pemetrexed) revealed a median PFS of 9.5 months, median OS of 20.7 months and an ORR of 34.6% [36] (Figure 2 and Supplementary Table S4). Another retrospective chart review of any-line pemetrexed in 121 ALK-positive and 266 ALK-negative patients (79 ALK-negative patients had Kirsten rat sarcoma (KRAS) virus proto-oncogene mutations and 187 had wild-type KRAS) showed that, for first-line pemetrexed–platinum, median PFS was significantly longer (8.5 months) in ALK-positive (n = 56) than in ALK-negative KRAS-positive (n = 44; 4.1 months; *p* = 0.004) and ALK-negative KRAS-negative patients (n = 104; 5.4 months; *p* = 0.018) [37] (Supplementary Table S4).

However, a cohort study suggested no difference in PFS or ORR between ALK-positive and wild-type patients, or those with EGFR or KRAS mutations, after pemetrexed generally given as first-line combination therapy. For patients with ALK rearrangements (n = 52), EGFR mutations (n = 188), KRAS mutations (n = 34) or wild-type disease (n = 168), median PFS was 7.8, 5.3, 4.6, and 4.3 months, respectively; ORR was 22.2%, 20.0%, 11.1%, and 33.3%, respectively [38].

#### 3.1.2. Pemetrexed Versus Non-Pemetrexed Regimens

In a retrospective cohort study of ALK-positive patients receiving first-line pemetrexed-based chemotherapy (n = 48) or other cytotoxic chemotherapy regimens (n = 78), median PFS was significantly longer with pemetrexed than with non-pemetrexed regimens (6.6 versus 3.8 months; *p* = 0.001) (Supplementary Table S4). In a multivariate analysis, only non-pemetrexed treatment significantly predicted poorer PFS (HR 1.91; *p* = 0.002). ORR (44.7 versus 14.3%; *p* < 0.001), but not OS (66.5 versus 49.2 months (NS)), was also significantly greater with pemetrexed than with non-pemetrexed regimens [39].

#### 3.1.3. Pemetrexed–Platinum Versus TKIs

Although first-line pemetrexed–platinum therapy provides survival benefit in ALK-positive patients, survival outcomes are longer with TKIs (e.g., crizotinib and ceritinib). In a phase III open-label study (PROFILE 1014) of 343 ALK-positive patients receiving first-line pemetrexed–cisplatin/carboplatin (n = 171), or crizotinib (n = 172), median PFS was 7.0 months with pemetrexed-based chemotherapy versus 10.9 months with crizotinib (HR 0.45; *p* < 0.0001). Similarly, ORR was 45% versus 74% (*p* < 0.001). At the time of analysis, there was no significant difference in OS or the probability of 1-year survival (Supplementary Table S4) [40].

In another phase III open-label study (ASCEND-4) of 376 ALK-positive patients receiving first-line pemetrexed–cisplatin/carboplatin then maintenance pemetrexed (n = 187), or ceritinib (n = 189), median PFS was 8.1 months with chemotherapy and 16.6 months with ceritinib (HR 0.55; *p* < 0.00001). Median OS (immature data) was 26.2 months versus not reached (HR 0.73; *p* = 0.056). Corresponding values for ORR were 26.7 versus 72.5% [41].

### 3.2. Second- and Later-Line Treatment

Pemetrexed monotherapy was effective for second- or later-line treatment of ALK-positive NSCLC. Results conflicted for whether pemetrexed was more effective in ALK-positive patients than in those with wild-type disease or other types of mutation but, overall, they favored greater efficacy in ALK-positive patients. In a cohort study of ALK-positive (n = 15), EGFR-positive (n = 43), or wild-type patients (n = 37) receiving second- or later-line pemetrexed monotherapy, ORR was significantly higher in ALK-positive (46.7%) than in EGFR-positive (4.7%) or wild-type patients (16.2%; *p* = 0.001) (Supplementary Table S4). For second-line pemetrexed only (n = 38), ORR was numerically but not significantly higher in ALK-positive than in EGFR-positive and wild-type patients (50.0%, 0%, and 19.2%, respectively); for third- or later-line treatment (n = 57), ORR was significantly higher in ALK-positive patients (44.4%, 5.4%, and 9.1%, respectively; *p* = 0.006). In a multivariate analysis, only ALK positivity significantly predicted a favorable ORR (HR 0.07; *p* = 0.001) [42].

In a previously mentioned study [37] in 121 ALK-positive patients and 266 ALK-negative EGFR wild-type patients, including 79 with KRAS mutations and 187 with wild-type KRAS, in which patients received any-line pemetrexed-based chemotherapy, median PFS did not differ significantly between ALK-positive (4.4 months), ALK-negative KRAS-positive (7.8 months), and ALK-negative KRAS-negative patients (3.8 months) receiving second- or third-line pemetrexed monotherapy (n = 31) (Supplementary Table S4).

Another previously mentioned cohort study [38] of ALK-positive (n = 52), EGFR-positive (n = 188), KRAS-positive (n = 34), and wild-type (n = 168) patients receiving any-line pemetrexed-based therapy, revealed significantly longer median PFS in ALK-positive patients than in all other mutation groups (8.7, 2.0, 1.6, and 1.9 months, respectively; *p* < 0.001) (Supplementary Table S4) for those receiving mainly second- or later-line pemetrexed monotherapy. A similar pattern was seen with ORR (29.0%, 8.4%, 8.7%, and 11.8%, respectively; *p* = 0.013).

#### Pemetrexed–Docetaxel Versus TKIs

In a phase III, open-label crossover study of 347 ALK-positive patients who progressed on one prior first-line platinum-based regimen, second-line crizotinib (n = 173) produced greater benefit than pemetrexed or docetaxel (n = 174). Median PFS was 3.0 months with chemotherapy and 7.7 months with crizotinib (HR for disease progression or death with crizotinib was 0.49 versus chemotherapy, 0.59 versus pemetrexed, and 0.30 versus docetaxel; *p* < 0.001 for all comparisons) (Supplementary Table S4). ORR was 20% with chemotherapy and 65% with crizotinib (intention-to-treat (ITT) population; *p* < 0.001), and 29%, 7%, and 66% with pemetrexed, docetaxel, and crizotinib, respectively (as-treated population; *p* < 0.001 for crizotinib versus both). Median OS was similar for chemotherapy and crizotinib (22.8 versus 20.3 months, respectively; HR 1.02; NS). This lack of survival benefit with crizotinib probably reflected the confounding effects of crossover [43].

### 3.3. Any-Line Treatment

Several studies compared any-line pemetrexed (combination or monotherapy) between patients with ALK rearrangements, EGFR or KRAS mutations, or wild-type disease. Results generally showed that any-line pemetrexed improved PFS and ORR in ALK-positive patients versus patients with other mutation types or wild-type disease.

In a previously mentioned study [37] of ALK-positive (n = 121) and ALK-negative EGFR wild-type patients (n = 266), including 79 with KRAS mutations and 187 with wild-type KRAS, patients received any-line pemetrexed-based chemotherapy as either combination or monotherapy. For patients receiving pemetrexed–platinum in any setting, median PFS was significantly longer in ALK-positive (7.3 months) than in ALK-negative KRAS-positive patients (4.5 months; *p* = 0.042) but was similar to that in ALK-negative KRAS-wild-type patients (5.9 months) (Supplementary Table S4). ALK-positive patients showed a trend toward improved median PFS when receiving bevacizumab as part of either the pemetrexed–platinum combination or maintenance therapy (9.5 versus 5.5 months without bevacizumab; *p* = 0.087). For patients receiving pemetrexed monotherapy or non-platinum–pemetrexed, median PFS was similar for ALK-positive (5.5 months), ALK-negative KRAS-positive (7.8 months), and ALK-negative KRAS-negative patients (3.9 months).

In a previously mentioned cohort study [38] of ALK-positive (n = 52), EGFR-positive (n = 188), KRAS-positive (n = 34), and wild-type (n = 168) patients receiving any-line pemetrexed as combination (mainly first-line) or monotherapy (mainly later-line), median PFS was significantly longer in ALK-positive (7.8 months) than in EGFR-positive (2.5 months), KRAS-positive (2.3 months), or wild-type patients (2.9 months) (*p* < 0.001) for all pemetrexed regimens combined (Supplementary Table S4). In a multivariate analysis, PFS was longer in ALK-positive than in other patients (HR 0.39 versus EGFR mutations, 0.42 versus KRAS mutations, 0.43 versus wild-type disease; *p* < 0.001 for all comparisons). ORR was also significantly higher in ALK-positive (26.9%) than in EGFR-positive (12.8%), KRAS-positive (8.8%), and wild-type (18.6%; *p* = 0.046) patients for all pemetrexed regimens combined.

In a retrospective review of ALK-positive (n = 19), EGFR-positive (n = 12), KRAS-positive (n = 21) or triple-negative (n = 37) patients who received any-line pemetrexed as combination or monotherapy, median PFS was longer for ALK-positive (9 months) than for EGFR-positive (5.5 months), KRAS-positive (7 months), or triple-negative patients (4 months) (Supplementary Table S4). In a multivariate analysis, only ALK positivity predicted prolonged PFS on pemetrexed (HR 0.36; *p* = 0.005). Line of therapy predicted worse PFS on pemetrexed (HR 1.57; *p* = 0.022). ORR was 42% for ALK-positive, 30% for EGFR-positive, 37% for KRAS-positive, and 14% for triple-negative patients [44].

## 4. ROS1 Rearrangements

Patients with ROS proto-oncogene1 (ROS1) rearrangement may benefit from pemetrexed-based treatment, with PFS similar to that in ALK-positive patients and longer than that in patients with echinoderm microtubule-associated protein-like 4-ALK (EML4-ALK), EGFR or KRAS mutations, or wild-type disease (Supplementary Table S5). In a cohort study comparing pemetrexed-based first-line therapy in ROS1-positive (n = 12), ALK-positive (n = 27), EGFR-positive (n = 34), KRAS-positive (n = 22) or quadruple-negative patients (n = 27), median PFS in ROS1-positive patients was similar to that in ALK-positive patients (6.8 and 6.7 months, respectively) and longer than that in EGFR-positive, KRAS-positive, or quadruple-negative patients (5.2, 4.2, and 4.5 months, respectively; among-group difference *p* = 0.003) [45].

A retrospective chart review of ROS1-positive (n = 5), ALK-positive (n = 13), and ROS1/ALK-negative patients (n = 144) receiving second- or third-line pemetrexed following first-line platinum-based therapy showed longer median PFS in ROS1-positive (not reached) than in ALK-positive (11.5 months) and ROS1/ALK-negative patients (3.3 months; *p* = 0.008 for ROS1-positive versus ROS1/ALK-negative patients) (Supplementary Table S5). ORR showed a similar pattern (60.0% for ROS1-positive versus 33.3% for ALK-positive (NS) and 8.5% for ROS1/ALK-negative patients (*p* = 0.01 for ROS1-positive versus ROS1/ALK-negative patients)). In Cox regression analysis, ROS1 rearrangement strongly predicted longer median PFS (HR 0.09; *p* = 0.02) [46].

**Figure 3:**
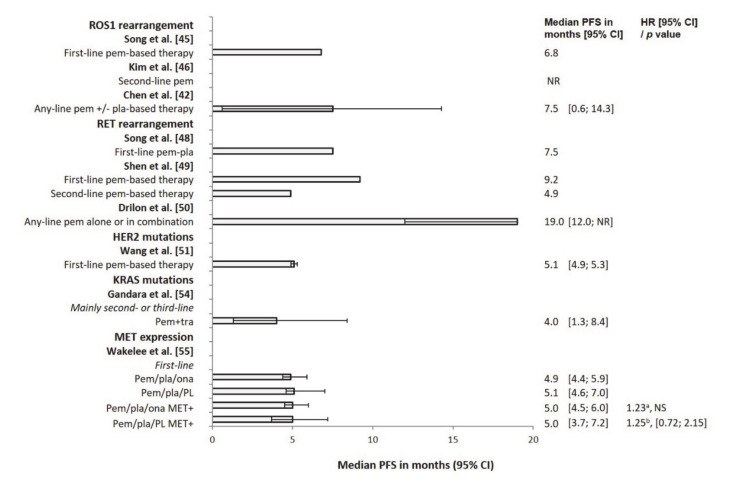
Median PFS with pemetrexed-based treatment in patients with advanced nonsquamous NSCLC and ROS1 or RET rearrangements, or HER2 or KRAS mutations, or MET expression. ^a^ Stratified HR. ^b^ Unstratified HR. Abbreviations: confidence interval (CI); human epidermal growth factor receptor 2 (HER2); Kirsten rat sarcoma virus proto-oncogene (KRAS); MNNG HOS-transforming (MET); non-small-cell lung cancer (NSCLC); neurotrophic tropomyosin receptor kinase (NTRK); programmed death ligand 1 (PD-L1); rearranged during transfection proto-oncogene (RET); ROS proto-oncogene 1 (ROS1); hazard ratio (HR); not reached (NR); not significant (NS); onartuzumab (ona); pemetrexed (pem); progression-free survival (PFS); placebo (PL); platinum (pla); rearranged during transfection proto-oncogene (RET); trametinib (tra).

In a retrospective cohort study of patients with ROS1 (n = 19), EGFR (n = 102), EML4–ALK (n = 32), or KRAS (n = 3) mutations/rearrangements, or quadruple-negative status (n = 97) who received any-line pemetrexed, as either combination or monotherapy, median PFS was longer in ROS1-positive (7.5 months) (Figure 3) than in EGFR-positive (3.7 months), EML4–ALK-positive (5.4 months), or quadruple-negative patients (4.1 months) (log-rank *p* = 0.003 for ROS1-positive versus other groups) (Supplementary Table S5). In a multivariate analysis, ROS1 positivity predicted longer PFS (HR 0.44; *p* = 0.005). ORR was significantly greater for ROS1-positive than for EGFR, EML4–ALK, and quadruple-negative patients (57.9%, 25.5%, 28.1%, and 24.7%, respectively; *p* = 0.026 for ROS1-positive versus other groups) (Supplementary Table S5) [47].

## 5. RET Rearrangements

Patients with rearranged during transfection (RET) proto-oncogene rearrangement may benefit from pemetrexed-based treatment, with median PFS similar to that in patients with ROS1 or ALK rearrangements and longer than in patients treated with other chemotherapy regimens and patients with KRAS-positive or wild-type disease. In a cohort study of four RET-positive and 64 RET-negative patients receiving first-line pemetrexed–platinum, median PFS was significantly longer in RET-positive than in RET-negative patients (7.5 versus 5.0 months; *p* = 0.026) (Supplementary Table S5). However, OS did not differ significantly between the two groups (58.1 versus 52.0 months) [48].

A retrospective review of 62 RET-positive patients showed that, for patients receiving first-line therapy (n = 40), median PFS was significantly longer in those treated with pemetrexed-based regimens (9.2 months) than in those treated with other chemotherapy regimens (paclitaxel-platinum or gemcitabine-platinum: 5.2 months; *p* = 0.007) (Supplementary Table S5). Corresponding values for second-line treatment (n = 28) were 4.9 and 2.8 months, respectively (*p* = 0.049). There was a trend towards longer OS in the pemetrexed-based treatment group (35.2 versus 22.6 months; *p* = 0.052) [49].

Another retrospective review of patients with a RET (n = 18), ROS1 (n = 10), or ALK (n = 36) rearrangement, or a KRAS mutation (n = 40) receiving any-line pemetrexed as combination or monotherapy revealed that median PFS was comparable for RET- (19 months), ROS1- (23 months) and ALK-positive patients (19 months) and significantly shorter in KRAS-positive patients (6 months; *p* = 0.005 versus RET, *p* = 0.002 versus ROS1, and *p* < 0.001 versus ALK). Median OS and ORR showed similar patterns (Supplementary Table S5) [50].

## 6. HER2 Mutations

Results with pemetrexed-based therapy in patients with Erb-b2 receptor tyrosine kinase (ERBB2, also known as human epidermal growth factor receptor 2 (HER2)) mutations are only available from observational studies, but have been favorable. A cohort study suggested that PFS and ORR in patients with HER2 mutations receiving first-line pemetrexed-based treatment were similar to those in patients with EGFR or KRAS mutations, but inferior to those in patients with ALK/ROS1 rearrangements. In the study, median PFS was 5.1 months in patients with HER2 mutations versus 6.5, 9.2, and 5.0 months in patients with EGFR, ALK/ROS1, and KRAS mutations, respectively. ORR was 36.0%, 33.8%, 41.3%, and 35.0%, respectively (Supplementary Table S5) [51].

Numerically longer treatment duration (indicating patients did not progress as quickly) was observed with pemetrexed-based first-line therapy than with other therapies in HER2-positive patients. However, results with later-line treatment have been conflicting. In a retrospective chart review of 38 patients with HER2 mutations receiving first- or later-line chemotherapy or HER2-targeted therapy, median duration of first-line treatment was longer with pemetrexed-based regimens (8.8 months (18 treatments)) than with taxane-based (4.0 months (five treatments)) or gemcitabine-based regimens (4.4 months (three treatments)) or HER2-targeted therapy (5.2 months (nine treatments)) (Supplementary Table S5). However, the differences were not statistically significant, and this pattern was not observed with later-line treatment (3.9, 4.0, 2.3, and 1.8 months, respectively) [52].

Similarly, an analysis of treatment duration in 29 patients with HER2 mutations receiving various first- or later-line treatment regimens showed that median duration of treatment was longer with first-line pemetrexed-based regimens (6.3 months) than with taxane- (5.3 months), gemcitabine- (4.9 months), or navelbine-based regimens (2.2 months) or gefitinib/erlotinib (2.1 months). A similar pattern was observed for duration of later-line treatment (6.0, 3.2, 5.4, 3.3, and 2.1 months, respectively) (Supplementary Table S5). In a multivariate analysis of the total study population (n = 591 with HER2, EGFR, ALK, or KRAS mutations), pemetrexed significantly predicted favorable OS (HR 0.48; *p* < 0.001) [53].

## 7. KRAS Mutations

Patients with a KRAS mutation may benefit from pemetrexed-based treatment, since the ORR with such treatment in one study exceeded that in patients with wild-type disease. However, this benefit was not reflected in median PFS. In this phase Ib open-label study comparing any-line pemetrexed–trametinib with docetaxel–trametinib in KRAS-positive (n = 48) or KRAS-negative/KRAS status unknown patients (n = 41), median PFS was shorter in KRAS-positive than in KRAS-negative/KRAS status unknown patients for pemetrexed–trametinib (4.0 versus 5.8 months; n = 23 and n = 19 for KRAS-negative/KRAS status unknown, respectively) and docetaxel–trametinib (3.4 versus 4.2 months; n = 25 and n = 22, respectively) (Supplementary Table S5). By contrast, ORR was numerically greater in KRAS-positive than in KRAS-negative/KRAS status unknown patients with both treatment regimens (17 versus 11% for pemetrexed–trametinib, 24 versus 18% for docetaxel–trametinib) [54].

## 8. MET Expression

In the control arm of a randomized study (GO27821), the survival benefit with pemetrexed-based therapy in patients with MNNG HOS transforming (MET) gene expression was similar to that in the overall NSCLC patient population for PFS but poorer for OS. Median PFS in MET-positive patients receiving first-line pemetrexed–platinum–placebo (n = 37) was 5.0 months, corresponding to that in the pemetrexed–platinum–placebo ITT population (n = 61:5.1 months) (Figure 3 and Supplementary Table S5) [55].

## 9. Future Perspectives

TKIs are the current standard of care for advanced NSCLC with targetable driver mutations. Since the addition of pemetrexed to gefitinib improves outcomes in patients with newly diagnosed EGFR-positive NSCLC [25,26], it is feasible that the addition of pemetrexed to the third-generation TKI osimertinib could also improve outcomes in such patients. A phase III open-label randomized study of first-line osimertinib with or without pemetrexed–platinum in approximately 556 patients with EGFR-positive advanced NSCLC (FLAURA2) is currently underway [58]. The study is expected to be completed in March 2026.

However, patients eventually develop resistance to TKIs [59,60] and treatment options after the failure of such therapy are limited. Immune checkpoint inhibitors that block programmed death-1/programmed death ligand 1 (PD-1/PD-L1), such as atezolizumab, nivolumab, and pembrolizumab, have recently been approved and recommended for the first- and second-line treatment of NSCLC without driver mutations [3,4,7,56]. However, inconsistent results have been obtained with these agents when given as monotherapy for second-line treatment in patients with EGFR-positive disease [61,62,63,64], and it therefore remains to be determined whether they are appropriate for use in patients with driver mutations. Combining immune checkpoint inhibitors with pemetrexed could improve outcomes [65], providing a potential therapeutic option following the development of TKI resistance. Indeed, several ongoing clinical studies are assessing the benefits of such combination treatment in patients with EGFR-positive advanced nonsquamous NSCLC who progressed after TKI therapy [66,67,68]. The studies CheckMate 722 [66] and WJOG8515L [67] are investigating nivolumab in combination with pemetrexed and a platinum, the KEYNOTE-789 study [68] is evaluating pembrolizumab in combination with pemetrexed and a platinum, and study NCT03924050 is evaluating toripalimab in combination with pemetrexed and a platinum. The results of these studies are awaited.

## 10. Conclusions

The identification of molecular mutations and rearrangements in NSCLC has been accompanied by the development of therapies targeting these alterations. However, cytotoxic agents such as pemetrexed still have an antitumor role in the treatment algorithm. Although guidelines generally recommend pemetrexed for first- and later-line treatment of advanced nonsquamous NSCLC in patients negative for gene mutations/rearrangements (i.e., with wild-type disease), this nonsystematic literature review suggests that survival outcomes with pemetrexed in patients with gene mutations/rearrangements are comparable to those in unselected patients. However, prospective clinical studies will be needed to confirm these findings.

Pemetrexed-containing regimens have been tested in first-line settings and as a sequential option following TKI failure in NSCLC patients with targetable driver mutations. In patients with EGFR, ALK, or ROS1 mutations, pemetrexed-based treatment improves PFS to a greater extent than in patients with wild-type disease, even after failure of TKI therapy. Limited data are available for patients with RET rearrangements or those with HER2 or KRAS mutations or MET expression. The few available studies suggest that in patients with RET rearrangements, the survival benefit with pemetrexed is similar to that in ALK- and ROS1-positive patients and longer than that in patients with KRAS mutations or wild-type disease. In patients with HER2 mutations, first-line pemetrexed-based therapy produces outcomes similar to those observed in patients with EGFR and KRAS alterations but shorter survival than in patients with ALK or ROS1 rearrangements. The ORR with pemetrexed-based therapy is greater in patients with KRAS mutations than in those with wild-type disease, but this benefit is not reflected in PFS. In patients expressing MET, pemetrexed-based therapy produces similar PFS to that in the overall NSCLC patient population, but shorter OS.

Possible reasons for differences in survival outcomes with pemetrexed across the different mutation types include different study methodology (some analyses lacked the power to assess certain endpoints), that a benefit in PFS may not translate into a benefit in OS due to the lack of post-discontinuation treatment options for some molecular alterations, differences in the patient populations studied due to differences in the methods used to identify molecular alterations, and that some molecular alterations may indicate a poor prognosis.

We suggest that chemotherapy with pemetrexed-based regimens still has a role in the treatment algorithm for patients with advanced nonsquamous NSCLC with targetable driver mutations and should not be overlooked as a treatment option in this era of targeted therapy.

## Figures and Tables

**Table 1:** Examples of genetic mutations/rearrangements identified in non-small-cell lung cancer (NSCLC) [3,4,5,6].

Genetic Mutation/Rearrangement	Examples of Targeted Therapy
EGFR gene mutations	Afatinib, erlotinib, gefitinib, osimertinib
ALK gene rearrangements	Alectinib, brigatinib, ceritinib, crizotinib, loratinib
RET gene rearrangements	Alectinib, selpercatinib
ROS1 rearrangements	Ceritinib, crizotinib, lorlatinib
ERBB2 (HER2) mutations	Afatinib, dacomitinib, trastuzumab
KRAS mutations	Selumetinib, trametinib
BRAF V600E point mutations	Dabrafenib + trametinib
PD-L1 expression	Atezolizumab, durvalumab, nivolumab, pembrolizumab
MET gene amplifications	Crizotinib
MET exon 14 skipping mutations	Cabozantinib, capmatinib, crizotinib
NTRK gene fusion	Entrectinib, larotrectinib

Abbreviations: anaplastic lymphoma kinase (ALK); B-Raf proto-oncogene (BRAF); epidermal growth factor receptor (EGFR); Er-b2 receptor tyrosine kinase 2 (ERBB2); human epidermal growth factor receptor 2 (HER2); Kirsten rat sarcoma (KRAS) virus proto-oncogene; MNNG HOS-transforming (MET); non-small-cell lung cancer (NSCLC); neurotrophic tropomyosin receptor kinase (NTRK); programmed death ligand 1 (PD-L1); rearranged during transfection proto-oncogene (RET); ROS proto-oncogene 1 (ROS1).

## Supplementary Materials

The following are available online at https://www.mdpi.com/2072-6694/12/9/2658/s1, Table S1: Summary of results from pivotal trials of pemetrexed in unselected patients with NSCLC, Table S2: Guideline recommendations for the use of pemetrexed in nonsquamous NSCLC, Table S3: Summary of results from studies of pemetrexed in patients with advanced nonsquamous NSCLC and EGFR gene mutations, Table S4: Summary of results from studies of pemetrexed in patients with advanced nonsquamous NSCLC and ALK rearrangements, Table S5: Summary of results from studies of pemetrexed in patients with advanced nonsquamous NSCLC and ROS or RET gene rearrangements, HER2 or KRAS mutations, or MET expression.
