# Supplementary material for: Does Pemetrexed Work in Targetable, Nonsquamous Non-Small-Cell Lung Cancer? A Narrative Review
Source: Cancers (Basel). 2020 Sep 17;12(9):2658. doi: 10.3390/cancers12092658 (PMC7563381; doi:10.3390/cancers12092658)
Supplement: Supplementary file 1 [file cancers-12-02658-s001.pdf]

Supplementary Table S1. Summary of results from pivotal trials of pemetrexed in unselected patients with NSCLC.

Study	Study design	Line of treatment	Pts	Treatment	Median PFS	Median OS	ORR
Scagliotti et al. [9]	Phase III noninferiority randomized study	First-line	N=1,725<br>Stage IIIB or IV NSCLC<br>Chemotherapy naïve	Cis (75 mg/m^2) + pem (500 mg/m^2) both on day 1 (n=862)<br>or<br>Cis (75 mg/m^2 on day 1) + gem (1,250 mg/m^2 on days 1 and 8; n=863)<br>Treatment every 3 wks for maximum of 6 cycles	**Overall:**<br>**Cis–pem:** 4.8 mo<br>**Cis–gem:** 5.1 mo<br>(HR 1.04; 95% CI 0.94–1.15)	**Overall:**<br>**Cis–pem:** 10.3 mo<br>**Cis–gem:** 10.3 mo<br>(HR 0.94; 95% CI 0.84–1.05).<br>**Nonsquamous NSCLC (n=1,000):**<br>**Cis–pem:** 11.8 mo<br>**Cis–gem:** 10.4 mo<br>(HR 0.81; 95% CI 0.70–0.94; p=0.005).<br>**Adenocarcinoma (n=847):**<br>**Cis–pem:** 12.6 mo<br>**Cis–gem:** 10.9 mo<br>(HR 0.84; 95% CI 0.71–0.99; p=0.03).<br>**Large-cell carcinoma (n=153):**<br>**Cis–pem:** 10.4 mo<br>**Cis–gem:** 6.7 mo<br>(HR 0.67; 95% CI 0.48–0.96; p=0.03).<br>**Squamous NSCLC (n=473):**<br>**Cis–pem:** 9.4 mo<br>**Cis–gem:** 10.8 mo<br>(HR 1.23; 95% CI 1.0–1.51; p=0.05)	**Overall:**<br>**Cis–pem:** 30.6%<br>**Cis–gem:** 28.2%

Paz-Ares et al. [10]	Phase III, multicenter, randomized, double-blind, PL-controlled study (PARAMOUNT)	Continuation maintenance	N=539<br>Stage IIIB or IV nonsquamous NSCLC<br>No progression after induction treatment with four 21-day cycles of pem–cis	Pem (500 mg/m²) + BSC (n=359)<br>or<br>PL + BSC (n=180)<br>Both on day 1 of 21-day cycles until disease progression, toxicity or discontinuation requested	**Pem:** 4.4 mo (95% CI 4.1–5.7).<br>**PL:** 2.8 mo (95% CI 2.6–3.0)<br>(HR 0.60; 95% CI 0.50–0.73; p<0.001)	**Pem:** 13.9 mo (95% CI 12.8–16.0).<br>**PL:** 11.0 mo (95% CI 10.0–12.5)<br>(HR 0.78; 95% CI 0.64–0.96; p=0.02)	
Ciuleanu et al. [11]	Phase III, multicenter, randomized, double-blind, PL-controlled study	Switch maintenance	N=663<br>Stage IIIB or IV NSCLC<br>No progression after induction treatment with four 21-day cycles of doublet chemotherapy (not including pem)	Pem (500 mg/m²) + BSC (n=441)<br>or<br>PL + BSC (n=222)<br>Both on day 1 every 3 wks until disease progression	**Overall:**<br>**Pem:** 4.3 mo (95% CI 4.1–4.7).<br>**PL:** 2.6 mo (95% CI 1.7–2.8)<br>(HR 0.50; 95% CI 0.42–0.61; p<0.0001).<br>**Nonsquamous (n=481):**<br>**Pem:** 4.5 mo (95% CI 4.2–5.6).<br>**PL:** 2.6 mo (95% CI 1.6–2.8)<br>(HR 0.44; 95% CI 0.36–0.55; p<0.0001).<br>**Squamous (n=182):**<br>**Pem:** 2.8 mo (95% CI 2.4–4.0).<br>**PL:** 2.6 mo (95% CI 1.6–3.2)<br>(HR 0.69; 95% CI 0.49–0.98; p=0.039)	**Overall:**<br>**Pem:** 13.4 mo (95% CI 11.9–15.9).<br>**PL:** 10.6 mo (95% CI 8.7–12.0)<br>(HR 0.79; 95% CI 0.65–0.95; p=0.012).<br>**Nonsquamous (n=481):**<br>**Pem:** 15.5 mo (95% CI 13.2–18.1).<br>**PL:** 10.3 mo (95% CI 8.1–12.0)<br>(HR 0.70; 95% CI 0.56–0.88; p=0.002).<br>**Squamous (n=182):**<br>**Pem:** 9.9 mo (95% CI 7.5–11.5).<br>**PL:** 10.8 mo (95% CI 8.5–13.2)<br>(HR 1.07; 95% CI 0.77–1.50; p=0.678)	**Overall:**<br>**Pem:** 6.8%<br>**PL:** 1.8%<br>(p=0.005)
Hanna et al. [12]	Phase III randomized study	Second-line	N=571<br>Stage III or IV NSCLC	Pem (500 mg/m²; n=283)<br>or	**Pem:** 2.9 mo<br>**Doc:** 2.9 mo	**Pem:** 8.3 mo<br>**Doc:** 7.9 mo	**Pem:** 9.1%

	One prior chemotherapy regimen	Doc (75 mg/m^2; n=288) Both on day 1 every 3 wks until disease progression, toxicity or discontinuation requested	(HR 0.97; 95% CI 0.82–1.16; p=0.759)	(HR 0.99; 95% CI 0.82–1.20; p=0.226)	**Doc:** 8.8% (p=0.105)

Abbreviations: BSC, best supportive care; cis, cisplatin; CI, confidence interval; doc, docetaxel; gem, gemcitabine; HR, hazard ratio; mo, months; NS, not significant; NSCLC, non-small-cell lung cancer; ORR, overall response rate; OS, overall survival; pem, pemetrexed; PFS, progression-free survival; pts, patients; PL, placebo; wks, weeks.

Supplementary Table S2. Guideline recommendations for the use of pemetrexed in nonsquamous NSCLC.

Guideline	Early-stage disease	Advanced disease	Maintenance treatment
NCCN [3]	**Post-operative treatment**<br>In combination with car in pts with high-risk, margin-negative stage IB disease who cannot tolerate cis	**Pts with PS 0–1 and negative or unknown test results for ALK rearrangements, sensitizing EGFR mutations or PD-L1 expression of <50% or unknown**<br>In combination with cis or car + pemb. If contraindications to pemb, with cis or car ± bev.<br>**Pts with PS 2 and negative or unknown test results for ALK rearrangements, sensitizing EGFR mutations or PD-L1 expression of <50% or unknown**<br>As single agent or in combination with car	**Continuation therapy‡**<br>As single agent or with pla/bev if given as the initial regimen.<br>**Switch therapy§**<br>As single agent in pts with negative or unknown test results for ALK rearrangements, sensitizing EGFR mutations or PD-L1 expression of <50% or unknown
ESMO [4,56]	**Stage I & II**<br>Adjuvant to surgery in combination with cis.<br>**Resectable & unresectable locally advanced**<br>In combination with cis	**First-line treatment in pts with PS 0–1 and EGFR-, ALK-, ROS1- or BRAF-negative disease**<br>In combination with cis or car + pemb or ate†, followed in combination with pemb or ate†; or in combination with cis or car ± bev.<br>**First-line treatment in pts with PS 2 & <70 years old and selected >70 years and PS 0–2 with EGFR-, ALK-, ROS1- or BRAF-negative disease**<br>As single agent or in combination with car.<br>**First-line treatment in pts with an EGFR mutation**<br>In combination with car and gef	**Continuation therapy‡**<br>As single agent following completion of 4–6 cycles of first-line pem-based treatment with no disease progression.<br>**Switch therapy§**<br>± bev
Pan-Asian (CSCO-ESMO) [7]		**First-line treatment in pts with no druggable oncogene driver**<br>Pemb + pem + pla if PS 0–1 and no contraindication to immunotherapy. Pla doublet (pem preferred to gem or doc) if contraindication to immunotherapy.<br>**Second-line treatment in pts with no druggable oncogene driver and contraindication to immunotherapy**<br>As single agent	**Continuation therapy‡**<br>**PS 0–1:** As single agent following 4 cycles of first-line pem–cis with no disease progression.<br>**Switch therapy§**<br>**PS 0–1:** As single agent ± bev following 4 cycles of first-line non-pem pla-based therapy with no disease progression.<br>**PS ≥2:** As single agent
ASCO [57]		**First-line treatment in pts with PS 0–1 and EGFR-, ALK-, or ROS1-negative disease**<br>In combination with cis or car.<br>**Second-line treatment in pts with PS 0–1 and EGFR-, ALK- or ROS1-negative disease**<br>As single agent if not previously given.	Not covered

**Third-line treatment in pts with PS 0–1 and EGFR-, ALK-, or ROS1-negative disease**
As single agent.
**Third-line treatment in pts who are EGFR positive and have received prior pla-based chemotherapy and an EGFR-TKI**
As single agent

[†]The combination of pem, car/cis and ate is not currently approved by the European Medicines Agency. [‡]Defined as the use of at least one agent given as first-line treatment. [§]Defined as the use of a different agent to those given as first-line treatment. Abbreviations: ALK, anaplastic lymphoma kinase gene; ASCO, American Society of Clinical Oncology; ate, atezolizumab; bev, bevacizumab; BRAF, B-Raf proto-oncogene; car, carboplatin; cis, cisplatin; CSCO, Chinese Society of Clinical Oncology; doc, docetaxel; EGFR, epidermal growth factor receptor gene; EGFR-TKI, EGFR-tyrosine kinase inhibitor; ESMO, European Society for Medical Oncology; gef, gefitinib; gem, gemcitabine; NCCN, National Comprehensive Cancer Network; NSCLC, non-small-cell lung cancer; PD-L1, programmed death ligand 1; pem, pemetrexed; pemb, pembrolizumab; pla, platinum; PS, performance status; pts, patients; ROS1, ROS proto-oncogene 1.

Supplementary Table S3. Summary of results from studies of pemetrexed in patients with advanced nonsquamous NSCLC and EGFR gene mutations.

Study	Study design	Line of treatment	Pts	Treatment	Median PFS	Median OS	ORR
**First-line treatment**							
**Pem + pla-based regimen**							
Jiang et al. [18]	Retrospective cohort study	First-line	EGFR mutation (n=69). Wild-type disease (n=89)	Pem–pla (n=96)	**EGFR-mutant:** 8.3 mo **Wild-type:** 6.7 mo (p=0.004)		**EGFR-mutant:** 43% **Wild-type:** 21% (p=0.039)
Kaneda et al. [19]	Multicenter retrospective medical record review	First-line	L858R (n=42). Del-19 (n=36). Wild-type (n=226)	Pem (500 mg/m^2) + cis (60–80 mg/m^2) every 3 wks for 4–6 cycles	**L858R:** 9.4 mo (95% CI 6.97–12.60). **Del-19:** 5.5 mo (95% CI 3.57–8.63; p=0.049 vs. L858R). **Wild-type:** 4.6 mo (95% CI 4.00–5.07). **EGFR mutation status predicted longer PFS:** HR 0.78 (95% CI 0.62–0.98; p=0.033)	**L858R:** 35.6 mo (95% CI 27.6–54.1). **Del-19:** 40.1 mo (95% CI 27.7–60.0; p=0.64)	**L858R:** 42.9% **Del-19:** 36.1% (p=0.21)
Noronha et al. [20]	Post-hoc analysis of a phase III, double-arm, parallel-group, open-label, randomized study	First-line	L858R (n=51). Del-19 (n=92)	Pem (500 mg/m^2) + car (AUC-5) every 3 wks for 6 cycles	**L858R:** 6.1 mo (95% CI 3.82–8.45). **Del-19:** 5.0 mo (95% CI 3.43–6.64; p=0.599) HR 0.90 (95% CI 0.62–1.32)	**L858R:** 18.1 mo (95% CI 13.5–22.6). **Del-19:** 24.5 mo (95% CI 21.3–27.7; p=0.002). **Type of EGFR mutation predicted longer OS:** HR 0.43 (95% CI 0.26–0.71; p=0.001)	**L858R:** 42.9% **Del-19:** 47.7% (p=0.706)
**Pem–pla vs. TKI**							

Patil et al. [21]	Open-label randomized parallel-group study	First-line	N=290	Pem (500 mg/m^2) + car (AUC-5) (n=145) followed by maintenance pem or Gef (250 mg daily) (n=145)	**Pem–car:** 5.6 mo (95% CI 4.2–7.0). **Gef:** 8.4 mo (95% CI 6.3–10.5) HR 0.66 (95% CI 0.51–0.85; p=0.001)	**Pem–car:** 22.6 mo (95% CI 18.6–26.6). **Gef:** 18.0 mo (95% CI 15.2–20.8) HR 1.28 (95% CI 0.92–1.79; p=0.133)	**Pem–car:** 45.3% **Gef:** 63.5% (p=0.003)
Sequist et al. [22] Yang et al. [23]	Phase III randomized study (LUX-Lung 3)	First-line	N=345	Pem + cis (standard doses) every 3 wks for up to 6 cycles (n=115) or Afa (40 mg/day; n=230)	**Independent review:** **Pem–cis:** 6.9 mo **Afa:** 11.1 mo HR 0.58 (95% CI 0.43–0.78; p=0.001). **Investigator review:** **Pem–cis:** 6.7 mo **Afa:** 11.1 mo HR 0.49 (95% CI 0.37–0.65; p=0.001). **For L858R or Del-19 only (n=308):** **Pem–cis:** 6.9 mo **Afa:** 13.6 mo HR 0.47 (95% CI 0.34–0.65; p=0.001) for independent review; HR 0.41 (95% CI 0.31–0.55; p=0.001) for investigator review	**Pem–cis:** 28.2 mo (95% CI 20.7–33.2). **Afa:** 28.2 mo (95% CI 24.6–33.6) HR 0.88 (95% CI 0.66–1.17; p=0.39)	**Independent review:** **Pem–cis:** 23% **Afa:** 56% (p=0.001). **Investigator review:** **Pem–cis:** 44% **Afa:** 69% (p=0.001)
Kato et al. [24]	Subgroup analysis of LUX-Lung 3 (32)	First-line	N=83, including: Del-19 (n=39), L858R (n=38)	Pem + cis (standard doses) every 3 wks for up to 6 cycles (n=29) or Afa (40 mg/day; n=54)	**Overall:** **Pem–cis:** 6.9 mo **Afa:** 13.8 mo HR 0.38 (95% CI 0.20–0.70; p=0.0014). **L858R or Del-19:** **Pem–cis:** 6.9 mo **Afa:** 13.8 mo HR 0.28 (95% CI 0.15–0.52; p<0.0001).	**Overall:** **Pem–cis:** 35.8 mo **Afa:** 46.9 mo HR 0.75 (95% CI 0.40–1.43; p=0.379). **L858R or Del-19:** **Pem–cis:** 35.0 mo **Afa:** 46.9 mo	**Overall:** **Pem–cis:** 20.7% **Afa:** 61.6% OR 6.52 (95% CI 2.22–19.14; p=0.0007). **L858R or Del-19:** **Pem–cis:** 22.2% **Afa:** 64.0% OR 6.22 (95% CI 2.12–18.24; p=0.0009).

					**L858R:**<br>**Pem–cis:** 8.3 mo<br>**Afa:** 13.7 mo<br>HR 0.50 (95% CI 0.20–1.25; p=0.131).<br>**Del-19:**<br>**Pem–cis:** 3.1 mo<br>**Afa:** 16.4 mo<br>HR 0.16 (95% CI 0.06–0.39; p<0.0001)	HR 0.57 (95% CI 0.29–1.12; p=0.097).<br>**L858R:**<br>**Pem–cis:** 40.3 mo<br>**Afa:** 41.7 mo<br>HR 1.13 (95% CI 0.40–3.21; p=0.821).<br>**Del-19:**<br>**Pem–cis:** 31.5 mo<br>**Afa:** 46.9 mo<br>HR 0.34 (95% CI 0.13–0.87; p=0.018)	**L858R:**<br>**Pem–cis:** 18.2%<br>**Afa:** 59.3%<br>OR 6.55 (95% CI 1.18–36.32; p=0.032).<br>**Del-19:**<br>**Pem–cis:** 25.0%<br>**Afa:** 69.6%<br>OR 6.86 (95% CI 1.63–28.90; p=0.0087)
**Pem or pem–pla + gef vs. gef**							
Cheng et al. [25]<br>Yang et al. [26]	Multicenter randomized phase II open-label study	First-line	N=191<br>Subgroup analysis according to Del-19 or L858R (n not given)	Pem (500 mg/m^2) every 3 wks + gef (250 mg daily) (n=126)<br>or<br>Gef (250 mg daily) (n=65)	**Overall:**<br>**Pem + gef:** 15.8 mo (95% CI 12.6–18.3).<br>**Gef:** 10.9 mo (95% CI 9.7–13.8).<br>Adjusted HR 0.68 (95% CI 0.48–0.96; one-sided p=0.014, two-sided p=0.029).<br>**Del-19:**<br>**Pem + gef:** 17.1 mo (95% CI 13.3–21.7).<br>**Gef:** 11.1 mo (95% CI 9.0–16.8).<br>HR 0.67 (95% CI 0.43–1.05; one-sided p=0.039, two-sided p=0.078).<br>**L858R:**<br>**Pem + gef:** 12.6 mo (95% CI 8.5–21.2).<br>**Gef:** 10.9 mo	**Pem + gef:** 43.4 mo (95% CI 33.4–50.8).<br>**Gef:** 36.8 mo (95% CI 26.7–42.6).<br>Adjusted HR 0.77 (95% CI 0.5–1.2; p=0.105)	**Overall:**<br>**Pem + gef:** 80%<br>**Gef:** 74%

					(95% CI 8.2–12.5).<br>HR 0.58 (95% CI 0.33–1.01;<br>one-sided p=0.027,<br>two-sided p=0.054)		
Seike et al. [27]	Randomized phase III open-label study	First-line	N=342	Pem (500 mg/m^2) + car (AUC-5) every 3 wks + gef (250 mg daily) (n=170)<br>or<br>Gef (250 mg daily) (n=172)	**Pem + car + gef:** 20.9 mo (95% CI 18.0–24.2).<br>**Gef:** 11.2 mo (95% CI 9.0–13.4).<br>HR 0.49 (95% CI 0.39–0.62; p<0.001)	**Pem + car + gef:** 52.2 mo<br>**Gef:** 38.8 mo<br>HR 0.70 (p=0.013)	
Noronha et al. [28]	Randomized phase III open-label study	First-line	N=350	Pem (500 mg/m^2) + car (AUC-5) every 3 wks + gef (250 mg daily) for 4 cycles (n=173)<br>or<br>Gef (250 mg daily) (n=177)	**Pem + car + gef:** 16.0 mo (95% CI 13.5–18.5).<br>**Gef:** 8.0 mo (95% CI 7.0–9.0).<br>HR 0.51 (95% CI 0.39–0.66; p<0.001)	**Pem + car + gef:** not reached.<br>**Gef:** 17.0 mo (95% CI 13.5–20.5).<br>HR 0.45 (95% CI 0.31–0.65; p<0.001)	**Radiologic response rate:**<br>**Pem + car + gef:** 75%<br>**Gef:** 63%<br>(p=0.01)
**Second- and later-line treatment**							
**Pem–pla or pem monotherapy**							
Jiang et al. [18]	Retrospective cohort study	Second- or later-line	EGFR mutation (n=69),<br>Wild-type disease (n=89)	Pem–pla (n=96),<br>Pem (n=62)	**Any-line doublet:**<br>**EGFR-mutant:** 7.5 mo<br>**Wild-type:** 6.4 mo (p=0.051).<br>**Any-line monotherapy:**<br>**EGFR-mutant:** 4.4 mo<br>**Wild-type:** 3.7 mo (p=0.115).<br>**Second- or third-line monotherapy:**<br>**EGFR-mutant:** 3.7 mo<br>**Wild-type:** 3.2 mo (p=0.078)		**Second-line doublet:**<br>**EGFR-mutant:** 27%<br>**Wild-type:** 13% (p=0.603).<br>**Second- or third-line monotherapy:**<br>**EGFR-mutant:** 13%<br>**Wild-type:** 8% (p=0.655)
Wu et al. [29]	Prospective cohort study	Second- or later-line (following	EGFR mutation (n=93), including:	Pem (500 mg/m^2) every 3 wks	**EGFR-mutant:** 3.9 mo (95% CI 3.1–4.7).<br>**Wild-type:** 2.3 mo	**EGFR-mutant:** 30.8 mo (95% CI 28.1–33.5).†	**EGFR-mutant:** 12.9%

		pem-based therapy)	Del-19 (n=43), L858R (n=37), Wild-type disease (n=63)		(95% CI 2.0–2.6; p=0.03 vs. EGFR-mutant). **L858R:** 4.0 mo (95% CI 3.3–4.7). **Del-19:** 3.3 mo (95% CI 1.6–5.0; p=0.641 vs. L858R). **EGFR mutation vs. wild-type was associated with PFS:** HR 0.68 (95% CI 0.49–0.94; p=0.021)	**Wild-type:** 25.8 mo (95% CI 17.7–33.9; p=0.439 vs. EGFR-mutant). **L858R:** 29.3 mo (95% CI 26.9–31.7). **Del-19:** 34.1 mo (95% CI 25.8–42.4; p=0.177 vs. L858R)	**Wild-type:** 1.6% (p=0.016 vs. EGFR-mutant). **L858R:** 10.8% **Del-19:** 14.0% (p=0.745 vs. L858R). **Classic mutation (Del-19 or L858R):** 12.5% **Nonclassic mutation:** 15.4% (p=0.673 vs. classic). **Second-, third- and fourth-line in EGFR-mutant:** 8.8%, 8.6%, 7.8% (p=0.980)
Joshi et al. [30]	Post-hoc analysis of a randomized study	Second-line (following first-line gef)	L858R (n=22), Del-19 (n=33)	Pem (500 mg/m^2) + car (AUC-5)	**L858R:** 4.77 mo (95% CI 1.37–8.16). **Del-19:** 5.90 mo (95% CI 4.27–7.53). HR 0.56 (95% CI 0.27–1.16; p=0.121)	**L858R:** 6.2 mo (95% CI 4.22–8.12). **Del-19:** 11.8 mo (95% CI 9.92–13.68; p=0.024). **Type of EGFR mutation did not predict OS:** HR 0.36; 95% CI 0.09–1.44; p=0.149)	**L858R:** 33.3% **Del-19:** 39.3% (p=0.752)
				**Pem–cis–gef vs. pem–cis–PL**			
Soria et al. [31]; Mok et al. [32]	Phase III multicenter randomized study (IMPRESS)	Second-line (following first-line gef)	N=265 T790M mutation-positive (n=142), T790M mutation-negative (n=105)	Pem–cis (500 mg/m^2 and 75 mg/m^2, respectively, every 3 wks) + gef (250 mg orally OD; n=133) or Pem–cis + PL (n=132) For ≤6 cycles	**Gef:** 5.4 mo (95% CI 4.5–5.7). **PL:** 5.4 mo (95% CI 4.6–5.5). HR 0.86 (95% CI 0.65–1.13; p=0.27). **T790M-positive:** **Gef:** 4.6 mo	**Gef:** 13.4 mo **PL:** 19.5 mo (HR 1.44; 95% CI 1.07–1.94; p=0.016). **T790M-positive:** **Gef:** 10.8 mo **PL:** 14.1 mo	**Gef:** 31.6% **PL:** 34.1% OR 0.92 (95% CI 0.55–1.55; p=0.76)

					**PL:** 5.3 mo<br>HR 0.97 (95% CI 0.67–1.42; p=0.883).<br>**T790M-negative:**<br>**Gef:** 6.7 mo<br>**PL:** 5.4 mo<br>HR 0.67 (95% CI 0.43–1.03; p=0.075)	HR 1.49 (95% CI 1.02–2.21; p=0.04).<br>**T790M-negative:**<br>**Gef:** 21.4 mo<br>**PL:** 22.5 mo<br>HR 1.15 (95% CI 0.68–1.94; p=0.609**)**	
				**Pem vs. non-pem regimen**			
Han et al. [33]	Systematic review of eight studies	Second-line (following TKI therapy)	1,193 pts, of whom 640 received pem	Pem monotherapy (three studies, 114 pts)<br>Pem–pla (seven studies, 526 pts)<br>Any pem (n=148) vs. non-pem (n=97; three studies)	**Any pem regimen:** weighted median PFS 5.1 mo.<br>**Non-pem regimen:** weighted median PFS 3.2 mo.<br>**Any pem vs. non-pem by study:**<br>4.2 vs. 2.7 mo (HR 0.54; 95% CI 0.34–0.86; p=0.009).<br>6.4 vs. 4.1 mo (HR 0.47; 95% CI 0.26–0.84; p=0.010).<br>4.7 vs. 3.3 mo (p=0.620)	**Any pem regimen:** weighted median OS 15.9 mo.<br>**Non-pem regimen:** weighted median OS 11.1 mo.<br>**Any pem vs. non-pem by study:**<br>15.1 vs. 11.0 mo (HR 0.92; 95% CI 0.50–1.68; p=0.785).<br>19.2 vs. 14.1 mo (HR 0.50; 95% CI 0.22–1.13; p=0.097).<br>15.1 vs. 8.1 mo (p=0.168)	**Any pem regimen:** weighted ORR 30.2%<br>**Non-pem regimen:** weighted ORR 18.3%<br>**Any pem vs. non-pem (two studies):** weighted ORR 30.2 vs. 18.3%<br>**Any pem vs. non-pem by study:**<br>32.4 vs. 17.4% (p=0.111)<br>26.0 vs. 20.0% (p=0.799)
Yang et al. [34]	Retrospective cohort study	Second-line (following first-line gef)	N=98	Erl (n=12)<br>Pem (n=2)<br>Gem (n=2)<br>Vin (n=21)<br>Tax (n=1)<br>Pem–pla (n=34)<br>Gem–pla (n=16)	**Pem–pla:** 6.4 mo<br>**Pla doublet no pem:** 4.1 mo (p=0.008 vs. pem–pla).<br>**Pem–pla vs. no pem doublet predicted longer PFS:** HR 0.42 (95% CI 0.23–0.77)	**Pem–pla:** 19.2 mo<br>**Pla doublet no pem:** 14.1 mo (p=0.164).<br>**Pem–pla vs. no pem doublet**	**Pem–pla:** 24%<br>**Pla doublet no pem:** 12% (p=0.234)

				Vin–pla (n=7)<br>Tax–pla (n=3)		**predicted longer OS:** HR 0.50 (95% CI 0.22–1.13; p=0.097)	
**Pem–pla vs. osi**							
Mok et al. [35]	Phase III, multicenter, randomized, open-label study (AURA3)	Second-line (following TKI therapy)	EGFR-T790M-positive disease (n=419)	Pem (500 mg/m^2) + car (AUC-5) or cis (75 mg/m^2) every 3 wks for ≤6 cycles<br>or<br>Osi (80 mg OD)	**Pem–car/cis:** 4.4 mo (95% CI 4.2–5.6).<br>**Osi:** 10.1 mo (95% CI 8.3–12.3).<br>HR 0.30 (95% CI 0.23–0.41; p<0.001)		**Pem–car/cis:** 31% (95% CI 24–40).<br>**Osi:** 71% (95% CI 65–76).<br>OR 5.39 (95% CI 3.47–8.48; p<0.001)

†In this study, OS was defined as the period from the date of first-line treatment to the date of death, last follow-up, or the final study follow-up day. Abbreviations: afa, afatinib; AUC, area under the concentration–time curve; car, carboplatin; cis, cisplatin; CI, confidence interval; CNS, central nervous system; Del-19, EGFR gene exon 19 deletion; EGFR, epidermal growth factor receptor; erl, erlotinib; gef, gefitinib; gem, gemcitabine; HR, hazard ratio; L858R, Leu858Arg point mutation in exon 21; mo, months; NSCLC, non-small-cell lung cancer; NS, not significant; OD, once daily; OR, odds ratio; ORR, overall response rate; OS, overall survival; osi, osimertinib; pem, pemetrexed; PFS, progression-free survival; PL, placebo; pla, platinum; pts, patients; tax, taxane; TKI, tyrosine kinase inhibitor; vin, vinorelbine; wks, weeks.

Supplementary Table S4. Summary of results from studies of pemetrexed in patients with advanced nonsquamous NSCLC and ALK rearrangements.

Study	Study design	Line of treatment	Pts	Treatment	Median PFS	Median OS	ORR
**First-line treatment**							
Pem + pla-based regimen							
Ma et al. [36]	Retrospective chart review	First-line	N=52	Pem (500 mg/m^2) alone or + cis (75 mg/m^2) or + car (AUC 4–5 mg/mL/min) every 3 wks	9.5 mo (95% CI 7.45–11.54)	20.7 mo (95% CI 14.13–27.33)	34.6%
Shaw et al. [37]	Multicenter retrospective chart review	Any-line (including first-line; prior first-line treatment for second- and later-line therapy not specified)	ALK-positive (n=121) ALK-negative (n=266), including 79 KRAS-positive and 187 KRAS-negative pts	Pem alone or as combination therapy	**First-line pem–pla**: **ALK-positive**: 8.5 mo (95% CI 5.9–10.9). **ALK-negative, KRAS-positive**: 4.1 mo (p=0.004 vs. ALK-positive). **ALK-negative, KRAS-negative**: 5.4 mo (p=0.018 vs. ALK-positive)		
Park et al. [38]	Cohort study	Any-line (including first-line, and second- and later-line following chemotherapy)	ALK-positive (n=52) EGFR-positive (n=188) KRAS-positive (n=34) Wild-type disease (n=168)	Pem alone or as combination therapy	**Pem combination (mainly first-line)**: **ALK-positive**: 7.8 mo **EGFR-positive**: 5.3 mo **KRAS-positive**: 4.6 mo **Wild-type**: 4.3 mo (p=0.227)		**Pem combination (first-line)**: **ALK-positive**: 22.2% **EGFR-positive**: 20.0% **KRAS-positive**: 11.1% **Wild-type**: 33.3% (p=0.448)
Pem- vs. non-pem regimens							
Jo et al. [39]	Retrospective cohort study	First-line	N=126	Pem-based chemotherapy (n=48) or non-pem-based chemotherapy (n=78)	**Pem**: 6.6 mo (95% CI 5.1–8.1). **Non-pem**: 3.8 mo	**Pem**: 66.5 mo **Non-pem**: 49.2 mo (p=0.919)	**Pem**: 44.7% **Non-pem**: 14.3% (p<0.001 vs. pem)

					(95% CI 2.8–4.8; p=0.001 vs. pem). **Non-pem associated with shorter PFS**: HR 1.91 (95% CI 1.27–2.87; p=0.002)		
			Pem–pla vs. TKIs				
Solomon et al. [40]	Phase III, open-label, randomized study (PROFILE 1014)	First-line	N=343	Pem (500 mg/m²) + cis (75 mg/m²) or car (AUC 5–6) every 3 wks for ≤6 cycles (n=171) or Cri (250 mg bid orally; n=172)	**Chemotherapy**: 7.0 mo (95% CI 6.8–8.2). **Cri**: 10.9 mo (95% CI 8.3–13.9). HR 0.45 (95% CI 0.35–0.60; p<0.001)	**HR for death with cri**: 0.82 (95% CI 0.54–1.26; p=0.36). **Probability of 1-year survival**: **Chemotherapy**: 79% (95% CI 71–84). **Cri**: 84% (95% CI 77–89). **HR for death with cri**: 0.60 (95% CI 0.27–1.42; Wilcoxon test); 0.67 (95% CI 0.28–1.48; log-rank test)	**Chemotherapy**: 45% (95% CI 37–53). **Cri**: 74% (95% CI 67–81; p<0.001)
Soria et al. [41]	Phase III open-label randomized study (ASCEND-4)	First-line	N=376	Pem (500 mg/m²) + cis (75 mg/m²) or car (AUC 5–6) every 3 wks for 4 cycles followed by maintenance pem (n=187) or Cer (750 mg/day; n=189)	**Pem–cis/car**: 8.1 mo (95% CI 5.8–11.1). **Cer:** 16.6 mo (95% CI 12.6–27.2) (HR 0.55; 95% CI 0.42–0.73; p<0.00001)	**Immature data: Pem–cis/car**: 26.2 mo (95% CI 22.8–not estimable). **Cer:** Not reached (95% CI 29.3–not estimable) (HR 0.73; 95% CI 0.50–1.08; p=0.056)	**Pem–cis/car**: 26.7% (95% CI 20.5–33.7). **Cer:** 72.5% (95% CI 65.5–78.7)
			**Second- or later-line treatment**				
Lee et al. [42]	Cohort study	Second- or later-line (following systemic chemotherapy,	ALK-positive (n=15)	Pem (500 mg/m²) every 3 wks			**Overall: ALK-positive**: 46.7%

		including a pla-based doublet)	EGFR-positive (n=43)<br>Wild-type disease (n=37)				**EGFR-positive**: 4.7%<br>**Wild-type**: 16.2% (p=0.001 for difference between the three groups).<br>**Second-line (n=38):**<br>**ALK-positive**: 50.0%<br>**EGFR-positive**: 0%<br>**Wild-type**: 19.2% (p=0.096 for difference between the three groups).<br>**≥Third-line (n=57):**<br>**ALK-positive**: 44.4%<br>**EGFR-positive**: 5.4%<br>**Wild-type**: 9.1% (p=0.006 for difference between the three groups).<br>**ALK associated with favorable ORR**: HR 0.07 (95% CI 0.01–0.32; p=0.001)
Shaw et al. [37]	Multicenter retrospective chart review	Any-line (including first-line; prior first-line treatment for second- and later-line therapy not specified)	ALK-positive (n=121)<br>ALK-negative (n=266), including 79 KRAS-positive and 187 KRAS-negative pts	Pem alone or as combination therapy	**Single-agent pem secondor third-line:**<br>**ALK-positive**: 4.4 mo (95% CI 2.1–9.0).<br>**ALK-negative, KRAS-positive**: 7.8 mo (p=0.606 vs. ALK-positive).		

					**ALK-negative, KRAS-negative**: 3.8 mo (p=0.787 vs. ALK-positive)		
Park et al. [38]	Cohort study	Any-line (including first-line, and second- and later-line following chemotherapy)	ALK-positive (n=52) EGFR-positive (n=188) KRAS-positive (n=34) Wild-type disease (n=168)	Pem alone or as combination therapy	**Pem monotherapy:** **ALK-positive**: 8.7 mo **EGFR-positive**: 2.0 mo **KRAS-positive**: 1.6 mo **Wild-type**: 1.9 mo (p<0.001). HR for ALK-positive vs. other groups: 0.39 vs. EGFR-positive, 0.42 vs. KRAS-positive, 0.43 vs. wild-type (p<0.001 for all comparisons)		**Pem monotherapy (second- or later-line):** **ALK-positive**: 29.0% **EGFR-positive**: 8.4% **KRAS-positive**: 8.7% **Wild-type**: 11.8% (p=0.013)
				Pem/doc vs. cri			
Shaw et al. [43]	Phase III open-label randomized study	Second-line (following pla-based chemotherapy)	347 ALK-positive pts who progressed on first-line pla-based therapy	Pem (500 mg/m^2) or doc (75 mg/m^2) every 3 wks (n=174) or Cri (250 mg orally bid; n=173)	**Pem/doc**: 3.0 mo (95% CI 2.6–4.3). **Cri**: 7.7 mo (95% CI 6.0–8.8). **HR for disease progression or death with cri:** 0.49 (95% CI 0.37–0.64; p<0.001) vs. pem/doc; 0.59 (95% CI 0.43–0.80; p<0.001) vs. pem; 0.30 (95% CI 0.21–0.43; p<0.001) vs. doc	**Pem/doc**: 22.8 mo (95% CI 18.6–not reached). **Cri**: 20.3 mo (95% CI 18.1–not reached). HR 1.02 (95% CI 0.68–1.54; p=0.54)	**ITT population:** **Pem/doc**: 20% (95% CI 14–26). **Cri**: 65% (95% CI 58–72; p<0.001). **As-treated population**: **Pem**: 29% (95% CI 21–39). **Doc**: 7% (95% CI 2–16). **Cri**: 66% (95% CI 58–73; p<0.001 vs. pem and vs. doc)
				**Any-line treatment**			
Shaw et al. [37]	Multicenter retrospective chart review	Any-line (including first-line; prior first-line treatment for second- and later-line therapy not specified)	ALK-positive (n=121) ALK-negative (n=266), including 79	Pem alone or as combination therapy	**Plat/pem in any-line setting:** **ALK-positive**: 7.3 mo (95% CI 5.5–9.5).		

			KRAS-positive and 187 KRAS-negative pts		**ALK-negative, KRAS-positive**: 4.5 mo (p=0.042 vs. ALK-positive). **ALK-negative, KRAS-negative**: 5.9 mo (p=0.182 vs. ALK-positive). **+ bev in combination or as part of maintenance therapy**: 9.5 vs. 5.5 mo for - bev (p=0.087). **Single-agent pem or non-pla/pem combination:** **ALK-positive**: 5.5 mo (95% CI 2.8–9.0). **ALK-negative, KRAS-positive**: 7.8 mo (p=0.860 vs. ALK-positive). **ALK-negative, KRAS-negative**: 3.9 mo (p=0.409 vs. ALK-positive)		
Park et al. [38]	Cohort study	Any-line (including first-line, and second- and later-line following chemotherapy)	ALK-positive (n=52) EGFR-positive (n=188) KRAS-positive (n=34) Wild-type disease (n=168)	Pem alone or as combination therapy	**Any pem regimen any-line:** **ALK-positive**: 7.8 mo **EGFR-positive**: 2.5 mo **KRAS-positive**: 2.3 mo **Wild-type**: 2.9 mo (p<0.001). **HR for ALK-positive vs. other groups**: 0.39 vs. EGFR-positive, 0.42 vs. KRAS-positive, 0.43 vs. wild-type (p<0.001 for all comparisons)		**Any pem regimen any-line:** **ALK-positive**: 26.9% **EGFR-positive**: 12.8% **KRAS-positive**: 8.8% **Wild-type**: 18.6% (p=0.046)
Camidge et al. [44]	Retrospective chart review	Any-line (including first-line; prior first-	N=89	Pem alone or as combination therapy	**ALK-positive**: 9 mo (95% CI 3–12).		**ALK-positive**: 42% **EGFR-positive**: 30%

	line treatment for second- and later-line therapy not specified)	ALK-positive (n=19)<br>EGFR-positive (n=12)<br>KRAS-positive (n=21)<br>Triple-negative (n=37)		**EGFR-positive**: 5.5 mo (95% CI 1–9).<br>**KRAS-positive**: 7 mo (95% CI 1.5–10).<br>**Triple-negative**: 4 mo (95% CI 3–5).<br>**Only ALK positivity associated with longer PFS**: HR 0.36 (95% CI 0.17–0.73; p=0.005).<br>**Line of therapy associated with worse PFS on pem**: HR 1.57 (95% CI 1.07–2.13; p=0.022)		**KRAS-positive**: 37%<br>**Triple-negative**: 14%

Abbreviations: ALK, anaplastic lymphoma kinase; AUC, area under the concentration–time curve; bev, bevacizumab; bid, twice daily; car, carboplatin; cer, ceritinib; CI, confidence interval; cis, cisplatin; cri, crizotinib; doc, docetaxel; EGFR, epidermal growth factor receptor; HR, hazard ratio; ITT, intention-to-treat; KRAS, Kirsten rat sarcoma virus proto-oncogene; min, minute; mo, months; NSCLC, non-small-cell lung cancer; ORR, overall response rate; OS, overall survival; pem, pemetrexed; PFS, progression-free survival; pla, platinum; pts, patients; TKI, tyrosine kinase inhibitor; wks, weeks.

Supplementary Table S5. Summary of results from studies of pemetrexed in patients with advanced nonsquamous NSCLC and ROS or RET gene rearrangements, HER2 or KRAS mutations, or MET expression.

Study	Study design	Line of treatment	Pts	Treatment	Median PFS	Median OS	ORR
**ROS1 rearrangement**							
Song et al. [45]	Cohort study	First-line	ROS1-positive (n=12)<br>ALK-positive (n=27)<br>EGFR-positive (n=34)<br>KRAS-positive (n=22)<br>Quadruple-negative (n=27)	Pem-based therapy	**ROS1-positive:** 6.8 mo<br>**ALK-positive:** 6.7 mo<br>**EGFR-positive:** 5.2 mo<br>**KRAS-positive:** 4.2 mo<br>**Quadruple-negative:** 4.5 mo (difference between groups p=0.003)		
Kim et al. [46]	Retrospective medical record review	Second-line (following first-line pla-based therapy)	N=162:<br>ROS1-positive (n=5)<br>ALK-positive (n=13)<br>ROS1/ALK-negative (n=144)	Pem	**ROS1-positive:** NR (p=0.008 vs. ROS1/ALK-negative).<br>**ALK-positive:** 11.5 mo<br>**ROS1/ALK-negative:** 3.3 mo<br>**ROS1 predicted longer median PFS:** HR 0.09 (p=0.02)		**ROS1-positive:** 60.0% (p=0.01 vs. ROS1/ALK-negative).<br>**ALK-positive:** 33.3% (p=0.12 vs. ROS1/ALK-negative).<br>**ROS1/ALK-negative:** 8.5%
Chen et al. [47]	Retrospective cohort study	Any-line (including first-line; prior first-line treatment for second- and	ROS1-positive (n=19)<br>EML4-ALK-positive (n=32)<br>EGFR-positive (n=102)	Pem alone or in combination with pla-based therapy	**Overall:**<br>**ROS1-positive:** 7.5 mo (95% CI 0.6–14.3; log-rank p=0.003 for		**Overall:**<br>ROS1-positive: 57.9% (p=0.026 for difference vs. other groups).

		later-line therapy not specified)	KRAS-positive (n=3) Quadruple-negative (n=97)		difference vs. other groups). **EGFR-positive:** 3.7 mo (95% CI 2.2–5.2). **EML4-ALK-positive:** 5.4 mo (95% CI 2.7–8.2). **Quadruple-negative:** 4.1 mo (95% CI 2.9–5.2). **ROS1-positivity associated with longer PFS:** HR 0.44 (95% CI 0.25–0.78; p=0.005). **EGFR-positivity associated with poorer PFS:** HR 1.43 (95% CI 1.04–1.96; p=0.028)		**EGFR-positive:** 25.5% **EML4-ALK-positive:** 28.1% **Quadruple-negative:** 24.7%
**RET rearrangement**							
Song et al. [48]	Cohort study	First-line	RET-positive (n=4) RET-negative (n=64)	Pem–pla	**RET-positive:** 7.5 mo **RET-negative:** 5.0 mo (p=0.026)	**RET-positive:** 58.1 mo **RET-negative:** 52.0 mo (p=0.504)	
Shen et al. [49]	Multicentre, retrospective study	First- or second-line	RET-positive (n=62)	**First-line:** Pem-pla (n=19) Pem monotherapy (n=3) Pac-pla (n=14) Gem-pla (n=4) **Second-line:** Pem-based (n=10) Other (n=18)	**First-line: Pem-based therapy:** 9.2 mo **Other chemotherap**y: 5.2 mo (p=0.007) HR 0.40 **Pem-based first-line treatment**	**Pem-based therapy (any line):** 35.2 mo **No pem:** 22.6 mo (p=0.052) HR 0.39	

					**predicted PFS:** p=0.008. **Second -line: Pem-based therapy:** 4.9 mo **Other chemotherapy:** 2.8 mo (p=0.049) HR 0.44		
Drilon et al. [50]	Retrospective chart review	Any-line (including first-line; prior first-line treatment for second- and later-line therapy not specified)	RET-positive (n=18) ROS1-positive (n=10) ALK-positive (n=36) KRAS-positive (n=40)	Pem alone or as combination therapy (pla or non-pla doublet ± bev)	**RET-positive:** 19 mo (95% CI 12–NR; p=0.005 vs. KRAS-positive). **ROS1-positive:** 23 mo (95% CI 14–NR; p=0.002 vs. KRAS-positive). **ALK-positive:** 19 mo (95% CI 15–36; p<0.001 vs. KRAS-positive). **KRAS-positive:** 6 mo (95% CI 5–9)	**RET-positive:** NR (95% CI 24–NR; p=0.004 vs. KRAS-positive). **ROS1-positive:** NR (95% CI 24–NR; p=0.08 vs. KRAS-positive). **ALK-positive:** 37 mo (95% CI 30–63; p<0.001 vs. KRAS-positive). **KRAS-positive:** 16 mo (95% CI 14–33)	**RET-positive:** 45% (p=0.30 vs. ROS1 and ALK-positive; p=0.39 vs. KRAS-positive). **ROS1-positive:** 78% **ALK-positive:** 50% **KRAS-positive:** 26% (overall comparison of the four subgroups p=0.02)
**HER2 mutations**							
Wang et al. [51]	Cohort study	First-line	HER2-positive (n=25) EGFR-positive (n=74) ALK/ROS-positive (n=39) KRAS-positive (n=40)	Pem-based therapy	**HER2-positive:** 5.1 mo (95% CI 4.90–5.30; p=0.247 vs. EGFR group; p=0.004 vs. ALK/ROS1 group; p=0.971 vs. KRAS group). **EGFR-positive:** 6.5 mo		**HER2-positive:** 36.0% **EGFR-positive:** 33.8% **ALK/ROS-positive:** 41.3% **KRAS-positive:** 35.0% (p=0.896 between groups)

					(95% CI 4.48–8.52). **ALK/ROS-positive:** 9.2 mo (95% CI 6.41–11.99). **KRAS-positive:** 5.0 mo (95% CI 3.67–6.33; p=0.971 vs. HER2 group; p=0.242 vs. EGFR group; p=0.007 vs. ALK/ROS1 group)
Eng et al. [52]	Retrospective medical record review	First- or later-line	N=38	Pem-based regimen (26 treatments) Tax-based (19 treatments) Gem-based (17 treatments) HER2-targeted (28 treatments)	**Median duration of first-line treatment:** **Pem-based:** 8.8 mo (range 1.3–21.0). **Tax-based:** 4.0 mo (range 0.8–20.2). **Gem-based:** 4.4 mo (range 2.5–11.5) **HER2-targeted:** 5.2 mo (range 1.3–16.3) (p=0.89 between groups). **Median duration of later-line treatment:** **Pem-based:** 3.9 mo (range 0–20). **Tax-based:** 4.0 mo (range 0.70–16.8). **Gem-based:** 2.3 mo (range 0–12). **HER2-targeted:** 1.8 mo (range 0.3–10)

Gow et al. [53]	Retrospective cohort study	First- or later-line	N=29	Pem-based regimen<br>(20 treatments)<br>Tax-based<br>(29 treatments)<br>Gem-based<br>(12 treatments)<br>Nav-based<br>(12 treatments)<br>Gef/erl<br>(22 treatments)	**Median duration of first-line treatment:**<br>**Pem-based:** 6.3 mo (range 2.3–17).<br>**Tax-based:** 5.3 mo (range 1.9–10.7).<br>**Gem-based:** 4.9 mo (range 2.6–7.2).<br>**Nav-based:** 2.2 mo (one treatment).<br>**Gef/erl:** 2.1 mo (range 1.0–11.7).<br>**Median duration of later-line treatment:**<br>**Pem-based:** 6.0 mo (range 1.3–37.4).<br>**Tax-based:** 3.2 mo (range 0.9–8.9).<br>**Gem-based:** 5.4 mo (range 0.3–36.6).<br>**Nav-based:** 3.3 mo (range 2.3–4.9).<br>**Gef/erl:** 2.1 mo (range 1.2–6.4)	**Pem associated with favorable OS:** HR 0.48 (95% CI 0.38–0.60; p<0.001)	
**KRAS mutations**							
Gandara et al. [54]	Phase Ib multicenter open-label nonrandomized study	Mainly second- or third-line (prior treatment not specified)	KRAS-positive (n=48)<br>KRAS-negative/KRAS status unknown (n=41)	Pem (500 mg/m^2 every 3 wks) + tra (1.5 mg daily) (n=42)<br>or<br>Doc (75 mg/m^2 every 3 wks) + tra (2.0 mg daily) (n=47)	**Pem + tra:**<br>**KRAS-positive:**<br>4.0 mo<br>(95% CI 1.3–8.4) (n=23).<br>**KRAS-negative/status unknown:**<br>5.8 mo		**Pem + tra:**<br>**KRAS-positive:**<br>17%<br>(95% CI 5.0–38.8).<br>**KRAS-negative/status unknown:** 11%<br>(95% CI 1.3–33.1).<br>**Doc + tra:**

					(95% CI 2.8–7.1) (n=19). **Doc + tra: KRAS-positive:** 3.4 mo (95% CI 1.5–6.3) (n=25). **KRAS-negative/status unknown:** 4.2 mo (95% CI 2.2–11.0) (n=22)		**KRAS-positive:** 24% (95% CI 9.4–45.1). **KRAS-negative/status unknown:** 18% (95% CI 5.2–40.3)
**MET expression**							
Wakelee et al. [55]	Phase II randomized multicenter double-blind PL-controlled study (GO27821)	First-line	N=259, of whom 73 were MET+	Pem/pla/ona (n=59, of whom 36 were MET+) Pem/pla/PL (n=61, of whom 37 were MET+) Bev/pac/pla/ona (n=69) Bev/pac/pla/PL (n=70). Results only presented for pem-based regimens	**Pem/pla/ona:** 4.9 mo (95% CI 4.4–5.9). **Pem/pla/PL:** 5.1 mo (95% CI 4.6–7.0). Stratified HR 1.23 (p=0.329). **Pem/pla/ona MET+:** 5.0 mo (95% CI 4.5–6.0). **Pem/pla/PL MET+:** 5.0 mo (95% CI 3.7–7.2) Unstratified HR 1.25 (95% CI 0.72–2.15)	**Pem/pla/ona:** 8.5 mo (95% CI 6.8–13.2). **Pem/pla/PL:** 13.7 mo (95% CI 6.6–not evaluable). Stratified HR 1.15 (p=0.591). **Pem/pla/ona MET+:** 8.0 mo (95% CI 6.8–13.2). **Pem/pla/PL MET+:** 7.6 mo (95% CI 5.9–not evaluable)	**Pem/pla/ona:** 28.6% **Pem/pla/PL:** 36.1% (p=0.389)

Abbreviations: ALK, anaplastic lymphoma kinase; bev, bevacizumab; CI, confidence interval; doc, docetaxel; EGFR, epidermal growth factor receptor; erl, erlotinib; gef, gefitinib; gem, gemcitabine; HER2, human epidermal growth factor receptor 2; HR, hazard ratio; KRAS, Kirsten rat sarcoma virus proto-oncogene; MET, tyrosine protein kinase; mo, months; nav, navelbine; NR, not reached; NSCLC, non-small-cell lung cancer; ona, onartuzumab; ORR, overall response rate; OS, overall survival; pac,

paclitaxel; pem, pemetrexed; PFS, progression-free survival; PL, placebo; pla, platinum; pts, patients; RET, rearranged during transfection proto-oncogene; ROS1, ROS proto-oncogene 1; tax, taxane; tra, trametinib; wks, weeks.

33
